# Application of Neural Network Models with Ultra-Small Samples to Optimize the Ultrasonic Consolidation Parameters for ‘PEI Adherend/Prepreg (CF-PEI Fabric)/PEI Adherend’ Lap Joints

**DOI:** 10.3390/polym16040451

**Published:** 2024-02-06

**Authors:** Dmitry Y. Stepanov, Defang Tian, Vladislav O. Alexenko, Sergey V. Panin, Dmitry G. Buslovich

**Affiliations:** 1 Laboratory of Mechanics of Polymer Composite Materials, Institute of Strength Physics and Materials Science of Siberian Branch of Russian Academy of Sciences, 634055 Tomsk, Russia; sdu@ispms.ru (D.Y.S.); vl.aleksenko@mail.ru (V.O.A.); 2 Department of Materials Science, Engineering School of Advanced Manufacturing Technologies, National Research Tomsk Polytechnic University, 634050 Tomsk, Russia; defan1@tpu.ru; 3 Laboratory of Nanobioengineering, Institute of Strength Physics and Materials Science of Siberian Branch of 9 Russian Academy of Sciences, 634055 Tomsk, Russia; buslovich@ispms.ru

**Keywords:** machine learning, neural network simulation, carbon fiber fabric, ultrasonic consolidation, lap joint, PEI, prepreg, interface, adhesion, structural integrity

## Abstract

The aim of this study was to optimize the ultrasonic consolidation (USC) parameters for ‘PEI adherend/Prepreg (CF-PEI fabric)/PEI adherend’ lap joints. For this purpose, artificial neural network (ANN) simulation was carried out. Two ANNs were trained using an ultra-small data sample, which did not provide acceptable predictive accuracy for the applied simulation methods. To solve this issue, it was proposed to artificially increase the learning sample by including additional data synthesized according to the knowledge and experience of experts. As a result, a relationship between the USC parameters and the functional characteristics of the lap joints was determined. The results of ANN simulation were successfully verified; the developed USC procedures were able to form a laminate with an even regular structure characterized by a minimum number of discontinuities and minimal damage to the consolidated components.

## 1. Introduction

Manufacturing processes, as a rule, are controlled according to several technological parameters, the combined influence of which determines the resulting product quality. Values of these parameters can individually exert opposite (conflicting) effects on each other, so they have to be optimized [1,2]. Solving such problems can be considered as a ‘classical’ design of experiment (DoE), and has attracted considerable attention from many researchers [3]. An example is turning, when it is necessary to simultaneously take into account the spindle speed, the feed rate, and a number of other factors [4]. In additive manufacturing by the fused filament fabrication/fused deposition modeling (FFF/FDM) method, both extruder and bed temperatures, the head speed, the material feed rate, and some additional parameters have different effects on the structure and properties of the 3D-printed products [5].

Since conducting a complete multifactorial experiment is not always possible (or rational), various optimization methods may be applied, for instance, the Taguchi [6] or Box–Behnken [7] techniques. Recently, artificial neural networks (ANNs) have increasingly begun to be used for solving such problems, especially for approximation or classification [8,9]. ANNs are characterized by high efficiency when a large (experimental) data sample is available [10]. However, the reliability of the prediction decreases (or it cannot be considered reliable at all) with limited data sets [11,12,13]. At the same time, numerous ANNs have been developed to date, so their correct selection and learning is a challenge under such conditions [14].

Ultrasonic welding (USW) of laminated polymer composites has been implemented in many high-tech industries (primarily aerospace) [15,16]. To form reliable welds, numerous USW parameters have to be optimized [17,18], including ultrasonic (US) frequency, amplitude of sonotrode vibration, clamping pressure, USW duration, duration of clamping after USW, etc. These parameters are the input data for the USW process [19], while its efficiency can be controlled by one output factor, namely the USW joint thinning, taking into account the need to insert an energy director (ED) between joined plates (adherends) [20].

It should be noted that USW can be used not only for joining laminates for structural components, but for the fabrication of laminates as well [21,22,23,24,25,26,27]. In such cases, their structure is formed due to processes developing at several interfaces, with the unilateral input of mechanical energy converted into frictional heating [28]. Respectively, the number of output parameters increases, since some physical, mechanical, dimensional, and structural characteristics have to be considered [29,30,31]. Their complete assessment is a rather long procedure, also requiring thorough statistical justification. The solution to this problem fully correlates with another production route for the formation of prepregs or laminates via automatic fiber placement assisted with heating via a laser beam [32,33,34,35,36,37].

A previous paper by the authors [38] was devoted to the optimization of the US-consolidation (USC) parameters for the formation of USC lap joints of polyetheretherketone (PEEK) adherends, a carbon fiber (CF) fabric prepreg impregnated with polyetherimide (PEI), and two PEEK EDs [39]. Impregnation of the CF fabric with the polymer, characterized by a low melting point and a melt flow index (MFI) greater than that for PEEK, determined the specific development of the structure formation process. In particular, molten PEI was squeezed out of the prepreg during USC, damaging the reinforcing CF fabric. Based on this experience, only PEI was utilized in this study, so PEI adherends were joined using the CF fabric impregnated with a PEI-based solution as well. A film of the low-melting TECAPEI (PEI-based copolymer) was inserted as an ED in the development of a USC procedure that firstly enables it to melt, ensuring the formation of lap joints with minimum possible damage to the CF fabric-based prepreg [30,40]. Hereinafter, these USC lap joints are designated as the ‘PEI adherend/Prepreg (CF-PEI fabric)/PEI adherend’ samples.

The aim of this study was to optimize the USC parameters by ANN simulation, providing the required functional characteristics of lap joints with a minimum number of full-scale experiments. To achieve this goal, it was firstly necessary to simulate the USC process as a ‘black box’ with many inputs and outputs. Then, two ANNs were trained using an ultra-small sample, which did not provide acceptable predictive accuracy for the applied simulation methods. It was proposed to implement a well-known approach, which consisted in artificially increasing the learning sample by including additional data synthesized according to the knowledge and experience of experts [41].

This paper is structured as follows. Section 1 provides an overview of the implementation of ANNs for optimization purposes, and generally reveals the ideology of their use to solve the problems highlighted in this study. Section 2 presents the sequence of ANN simulation of the USC process with an analysis of both a priori and a posteriori knowledge, as well as the obtained results, while Section 3 is devoted to their verification. Section 4 discusses the prospects for application of the developed approach using an example of laminated composites formed by layer-by-layer USC processing of the prepregs based on the CF fabric impregnated with the PEI solution. All the above results are summarized in conclusions.

A brief overview of the application of machine learning methods using small experimental data samples. As the authors have shown previously [42], the issue of finding an optimal combination of USC parameters cannot be considered as an optimization problem, since the conditions for ensuring the optimality of the functional characteristics of such lap joints are represented by a system of inequalities. This formulation is determined not only by the inconsistency of the requirements (formulation of criteria) for their individual properties, but also by the specifics of the problem. Practical interest is not in the specific values of the USC parameters, but their ranges. So, a solution to this problem should be approached in two stages: (i) approximation of a vector quantity (characteristics of a lap joint) in the multidimensional space of the USC parameters, and (ii) search for such a range of values of the parameter vector within which all the inequalities of the optimality condition are satisfied. In the general case, such areas contain an infinite number of solutions, so calling all of them ‘optimal’ (in the classical sense) is incorrect. More precisely, they should be referred to as areas of ‘suboptimal’ parameters (SOPs), within which the optimal solution is located.

The first stage of approximation can be carried out using (i) linear interpolation algorithms based on triangulation, (ii) inverse distance weighting or polynomial methods, (iii) basis function approach, (iv) Kriging interpolation, (v) piecewise linear function, and (vi) component-wise splines [43,44,45,46,47,48]. Each of these methods has both advantages and drawbacks. Nevertheless, only ANN simulation is recommended for universal approximation of a vector quantity in a multidimensional space, taking into account the relationship of the vector components and the significant non-linearity of the observed patterns [49].

The second stage of searching and constructing SOP areas based on the results of ANN simulation can be carried out using well-known methods of cluster analysis and image processing. The issue of the implementation of one of these is outside the scope of this study since it deserves a separate investigation.

One of the key challenges in ANN simulation is the selection of the ANN type and architecture. For processing static data, feedforward neural networks (FFNNs), radial basis function networks (RBFNN)s, and their modifications are most often used [50,51]. The numbers of layers and neurons in them are determined not only by the complexity of an approximated dependence but also by the learning sample size [52]. For small samples, the complexity of the applied model is typically neglected. In the presence of a large number of factors, reducing the dimension of the problem is achieved by highlighting one or two of the most significant factors, while the rest are not used for simulation. In [53], it was proposed to compensate for the simplification of ANNs by including additional parameters to the significant factors obtained using known output/input relationships. The complexity of ANNs depends on the numbers of their layers and neurons, as well as the justified relationships. In some cases, ANNs are divided into two or more interrelated (but simple) types for clarity. A special approach to the development of ANNs that considers the known physical laws of simulated processes is described in [54,55]. In that case, the ANN architecture was designed according to those laws and includes a hybrid physical–statistical learning method that explicitly embeds the solution of partial differential equations into the loss function of the so-called physics-informed neuron networks (PINNs).

Great attention is paid to numerous techniques for training ANNs, such as (i) learning algorithms for solving direct problems (for example, based on the finite element method), (ii) genetic algorithms, (iii) support vector machines [51,56], etc.

ANNs are characterized by the so-called ‘curse of dimensionality’: as the dimensions of their input vectors rise, the complexity of ANNs increases exponentially [49]. As a result, a learning sample has to be enhanced. For example, the following procedures should be implemented in one of the most common classes of problems related to classification and image recognition [57,58]: (i) generation of surrogate data, (ii) interpolation of experimental data, (iii) algorithms for shifting, permutation, reflection, and rotation of data to achieve system invariance, or (iv) randomization procedures to increase noise immunity.

Due to both the high labor intensity and the cost of full-scale research in materials science, the number of experiments performed is typically negligible [12,59,60]. In these cases, the authors mean by the ‘ultra-small sample’ concept such experimental data arrays that are sufficient to draw a linear or quasi-linear relationship, but significantly less than are necessary to formulate an adequate non-linear one. For example, the number of experiments can vary from nine (for the Taguchi method) to twenty-seven (for the fractional full factorial design) when designing an investigation with three factors and their levels. In such cases, the results of hundreds of experiments are required to train ANNs, but thousands are required for deep learning. Therefore, ANN simulation is characterized by high errors in the approximation region for ultra-small samples, no matter what type of ANN and training method are implemented. This is especially true outside the range of experimentally determined values. Such a phenomenon is referred to by many researchers as ‘the poor ability of ANNs to solve extrapolation problems’ [61]. It would seem an obvious and correct conclusion that using ultra-small learning samples requires abandoning the implementation of ANNs; however, they may be suitable for solving numerous applied problems that do not require high accuracy of simulation results, i.e., when a quality solution is enough.

**Figure 1:**
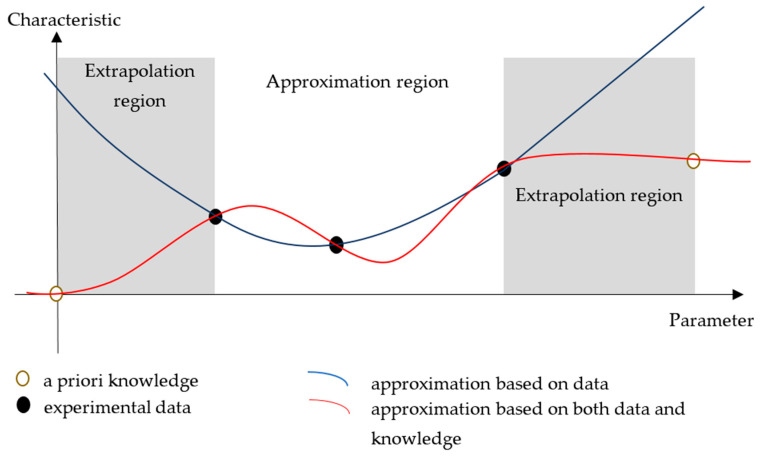
An example of changes in the results of one-dimensional approximation of experimental data with the additional use of a priori knowledge.

It should be noted that for ANN simulation of objects and processes that are not characterized by periodicity or high correlation of values in parameter spaces, the task of extrapolating the small experimental data samples cannot be solved correctly without additional information. Therefore, one of the possible ways to minimize errors in the approximation and extrapolation regions is to add a priori known data for the boundary, limit, or special parameter values (Figure 1). It is more correct to designate the latter a priori knowledge rather than data, because they are not obtained experimentally but are rather based on the experience of experts. A priori knowledge can be considered in a broad sense, since it includes both theoretical premises and previously comprehended knowledge about the simulated objects or processes. It is not always possible to formulate such knowledge, so the development of ANNs should be carried out in stages, assessing the adequacy of developed models and the accuracy of obtained results, as well as adding a posteriori knowledge, if necessary.

## 2. ANN Simulation with Ultra-Small Samples to Optimize the USC Parameters for the Formation of ‘PEI Adherend/Prepreg (CF-PEI Fabric)/PEI Adherend’ Lap Joints

### 2.1. A General Model of the USW Process

As mentioned above, conventional input (control) USC parameters include US frequency (*ω*), amplitude of sonotrode vibrations (*θ*), clamping pressure (*P*), USC duration (*t*), duration of clamping after USC (*τ*), etc. [38]. In this study, it was assumed that the values of the *t*, *τ*, and *P* parameters varied within specified ranges for optimizing the USC process, whereas the other two (*ω*, *θ*) were constant. This decision was based on the specifications of the deployed ‘UZPS-7’ ultrasonic welding machine (‘SpetsmashSonic’ LLC, Russia).

The following mechanical characteristics were used as the required properties of USC lap joints [38]:

(a) tensile strength (*σ*), MPa;
(b) elongation at break (*ε*), %;

as well as the dimensional parameters:

(c) USC joint thinning (Δ*d*), µm;
(d) top ED thickness, according to the image analysis (*δ*_ED top_), µm;
(e) distance between PEI adherends (*δ*_ED+CF_), µm;
(f) top PEI adherend integrity, assessed qualitatively (*C*), +/–.

**Figure 2:**
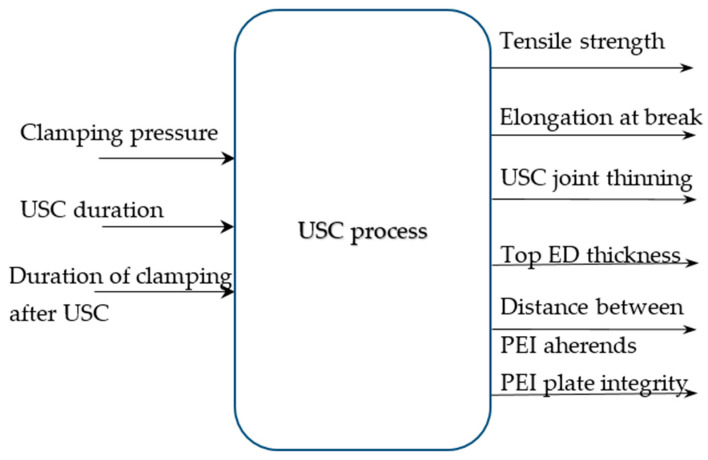
A general model of the USC process.

Considering that the USC process was a transformation of the properties of all components of the lap joints due to external impacts, and its law was significantly non-linear and unknown as well, the model of this process can be represented as a ‘black box’ with three inputs and six outputs (Figure 2).

### 2.2. ANN Architecture

**Figure 3:**
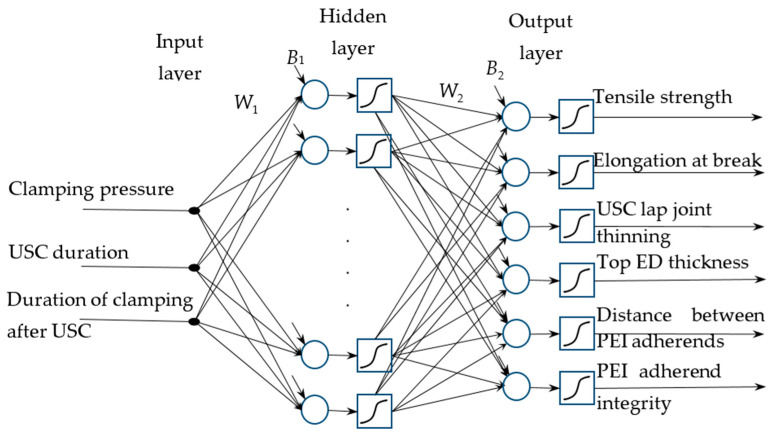
Architecture of the feedforward function for the FFNN: *W*_n_ are matrices of synaptic weights, *B*_n_ are vectors of bias values.

**Figure 4:**
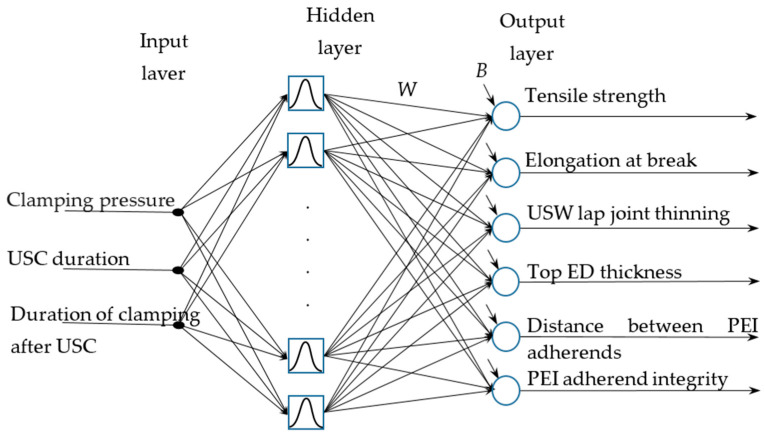
Architecture of the radial basis function for the RBFNN: *W*_n_ are matrices of synaptic weights, *B*_n_ are vectors of bias values.

In this study, both FFNN and RBFNN were implemented. According to Figure 2, the inputs were three USC parameters, while six outputs were the functional characteristics of the lap joints. In their feedforward architectures, one hidden neural layer was used (Figure 3). In the case of FFNN, the following parameters had to be studied: the number of neurons in the hidden layer, the type of activation function of each layer, the selection of both learning and testing samples, etc. The RBFN architecture was uniquely defined except for the number of neurons in the hidden layer (Figure 4).

It was determined by its learning algorithm, which possessed two parameters: the ‘spread’ (the radius of the response of neurons to the input stimulus/distance between the input data) and the ‘goal’ permissible error (between the approximation and the values of the learning sample). Both these parameters were determined from an analysis of the distances between the input data of the learning sample and the measurement errors of the output data.

Due to the different dimensions of the USC parameters and the functional characteristics of the lap joints, the values of both input and output data spaces were normalized to dimensionless before the simulation. As the obtained results show, the highest rate of convergence of the ANN learning algorithms was achieved when normalized to the [−1, 1] interval. Formal criteria for the quality of such models included a number of assessments of the difference between the test sample and the predicted values (standard deviation, mean square, determination coefficient, etc.) used in the ANN learning algorithms. However, according to the authors’ best knowledge, no formal criteria have so far been proposed to justify the adequacy and overlearning of ANNs.

### 2.3. Analysis of a Priori Knowledge and Its Implementation

A preliminary analysis of the USC parameters and the expected results was based on previously acquired knowledge about the process patterns:

1. USC procedures could not be successfully completed due to their short durations and/or insufficient or excessively high clamping pressures. In these cases, the dimensions of the USC lap joints were not changed, while the mechanical properties were minimal.
2. If the USC durations were too long, the components being joined were partially or completely damaged. The USC lap joint thicknesses were minimal (after their maximum thinning), while the mechanical properties were low (but above zero values).
3. Releasing the clamping pressure immediately after USC reduced the mechanical properties of the lap joints to below acceptable levels. Moreover, their dimensions predominantly depended on the USC parameters (duration, clamping pressure) at the first stage, while the mechanical properties were determined by both USC duration and clamping duration after USC.

Since it was not possible to simultaneously achieve extreme (maximum or minimum) values of all the functional characteristics of the USC lap joints, their acceptable ranges were specified, i.e., the optimality conditions (Table 1). The quantitative values of the range boundaries were based on the previous experience of the authors [38] and the results of an analysis of data reported by other researchers. Accordingly, further search for the optimal combination of the USC parameters was carried out taking into account the values given in Table 1.

Trial numerical investigations to determine the optimal USC parameters were performed using only the a priori knowledge (the first two features mentioned above). In this case, the ANN simulation was not enabled to achieve the goal due to patterns in the learning data structure. Separately, the parameter values were the maximum values of the acceptable ranges of the functional characteristics of the USC joints. Their approximation also gave similar levels that did not satisfy the optimality conditions. Therefore, subsequent learning samples included both a priori knowledge and predictive parameters within the acceptable ranges.

**Figure 5:**
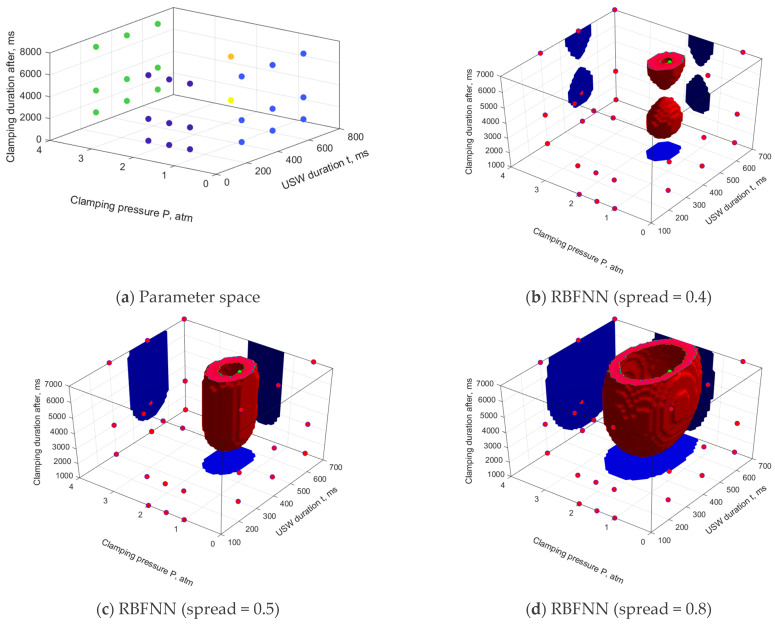

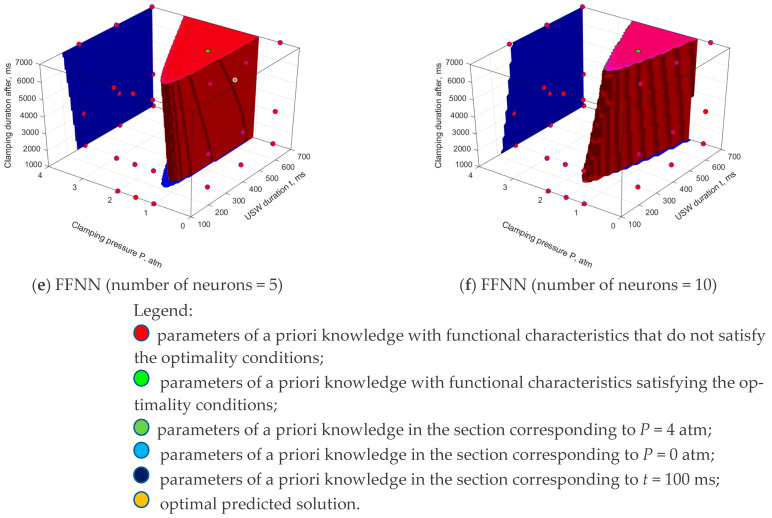
The USC parameters of a priori knowledge (**a**) and the SOP areas, constructed using the RBFNN (**b**–**d**) and FFNN (**e**,**f**) models. A priori knowledge data.

Advanced numerical investigations were based on a priori knowledge of the USC parameters at which such procedures could not be successfully completed and the levels required to achieve the optimal functional characteristics of the lap joints (Table 2). Figure 5 presents the results of the ANN simulation and assessment of the SOP area, drawn using the data from Table 2 when varying each range of the USC parameters presented in three rows for condition #2 “Constancy of dimensions’. The learning sample included 29 vectors of the USC parameters and the corresponding ones of the functional characteristics. In this case, the RBFNN (‘goal’ = 0.001) and FFNN (activation functions are hyperbolic tangents) were applied, with variation of their other parameters.

Note that the USC parameters presented in Table 2 limited the search area for their optimal values, but lay only in three sections of the corresponding space. As follows in Figure 5, this turned out to be enough to limit the SOP areas drawn using the RBFNN over their entire volumes. The ‘spread’ parameter exerted a direct impact on the sizes of the SOP areas: increasing the parameter gave rise to enlargement of the SOP areas. At this stage of the analysis using a priori knowledge, only models with ‘spread’ values below 0.5 were recognized as inadequate. In this case, it was accepted that the *τ* parameter was not dominant [38] and the SOP areas could not be discontinuous. Obviously, such a gap was associated with a great relative distance between the points of the predicted optimal USC parameters and could be eliminated by supplementing the sample with intermediate data. To the best of the authors’ knowledge, the problem of selection of an adequate value for the ‘spread’ parameter has not been formally solved for the RBFNN.

For the FFNN models, the absence of restrictions on three sections of the parameter space led to the unboundedness of the SOP areas in the direction of increasing the *t* parameter and in all directions of the *τ* parameter. It was noted that all the studied FFNN models implemented reliable approximation under these conditions but did not correspond to the a priori physical and mechanical concepts of the SOP areas. In this case, increasing the FFNN complexity (the number of neurons in the hidden layer) did not result in any significant changes in the SOP areas.

Additional a priori knowledge in the form of local points were added to the models as new variable parameters, namely the number of values and their distribution in space. Accordingly, it was necessary to use a uniform distribution of a priori knowledge in the parameter space according to Table 2, and to assess the influence of the number of additional points. In each of the three sections of the *τ* = 100 ms, *P* = 0.5 atm, and *P* = 4 atm space of the models described above, a priori knowledge was determined in the amounts of 6, 9, 12, and 18 points. The learning sample sizes were 20, 29, 38, and 56, respectively.

Subsequent numerical investigations included the development of a set of both RBNNs and FFNNs. The ‘spread’ value was 0.5, while the ‘goal’ levels varied from 10^–3^ to 10^–6^. The FFNNs were characterized by a single hidden layer (five neurons) and a log tangent activation function. Bayesian regularization was used as a learning algorithm.

**Figure 6:**
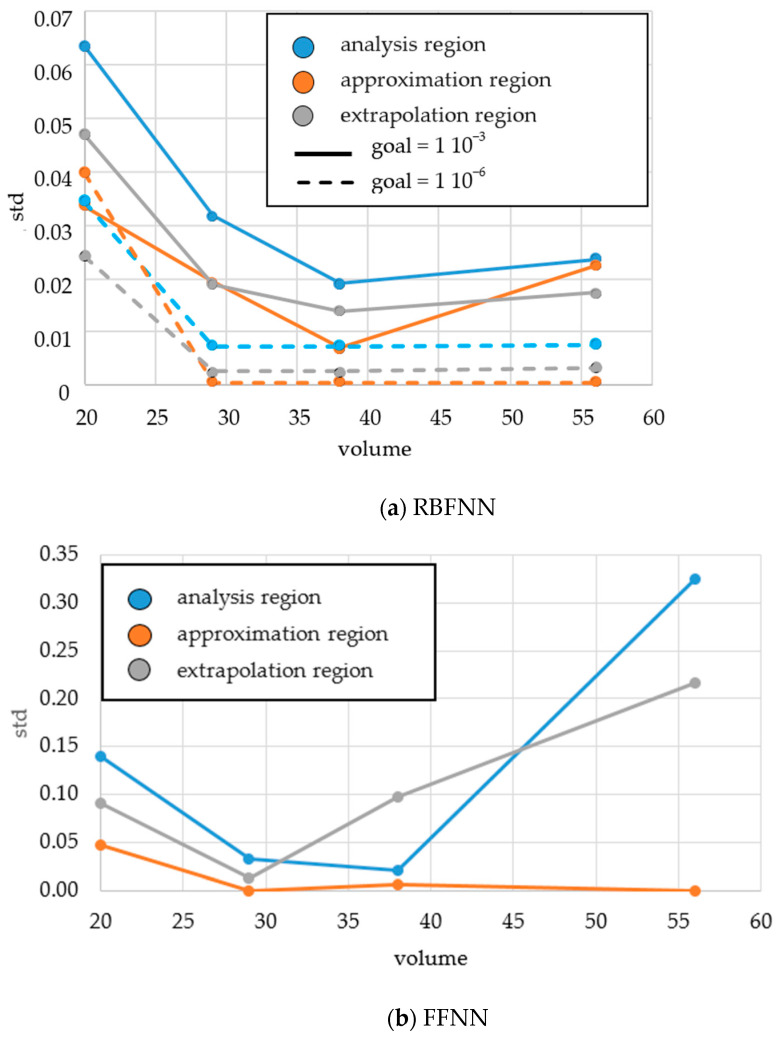
The dependences of the model accuracy on the sizes of a priori knowledge learning samples.

The accuracy of the models was assessed by the standard deviation of the data from the a priori values at the outputs. Errors were calculated for three cases: (i) the approximation region was limited to the predicted ranges of the optimal USC parameters, (ii) the extrapolation region was limited to the maximum predicted optimal USC parameters and the maximum boundaries of the acceptable ranges (*t* = 700 ms, *P* = 4 atm, *τ* = 9000 ms), and (iii) the scope of the analysis was limited to the acceptable ranges of the USC parameters. Since the FFNN learning results were multi-valued, the errors in these cases were estimated from the ensemble of synthesized and learned networks. Table 3 and Figure 6 show some of the results of calculating the errors of ANN simulation as instances.

As expected, the errors exhibit complex, ambiguous, and extreme patterns. According to Figure 6a and Table 3, for the RBNNs, the errors of the simulation depended on the ‘goal’ parameter as well as the sample size. The errors in the extrapolation and approximation ranges were comparable and repeated the behavior of the errors in the field of analysis. This indicates that the RBFNNs’ results might be validated only over the data of the latter error. For FFNNs, a substantial difference was found in the behavior of the error with an increase in the sample size. After reaching a minimum, the error in the approximation range changed insignificantly, while in the extrapolation range it began to drastically increase.

In the general case, determining the optimal size of the learning sample could not be formulated as ‘the higher, the better’ for both RBFNNs and FFNNs. This is related to the problem of redundancy of the training sample, which occurs with large sample sizes. However, the use of deep learning can result in decreasing the accuracy of the simulation [10]. This is usually solved by introducing one or more of various methods of reducing the dimensions of the source data or the total sample size. Among them are the principal component method, linear discriminant analysis, random or deterministic sampling, etc. [62,63,64,65]. In the current study, the optimal size of the training sample in terms of minimizing the error of approximation and extrapolation was addressed as the problem of searching for the minimum error. Obviously, this task was of the search type. For the data presented above, the best results of the ANN simulation were achieved with a learning sample size of 38 (9 additional points with a priori knowledge in each section of the parameter space).

Comparing the errors in the approximation and extrapolation regions with the numbers of neurons in the RBFNNs after learning, the following could be concluded. Their training algorithm enabled them to avoid overlearning the model, i.e., an increase in the sample size did not enhance the number of neurons in all cases. Perhaps for this reason, it also did not contribute to a significant increase in extrapolation errors. In contrast to the RBFNNs, increasing the learning sample size of the FFNNs could lead to a significant increase in extrapolation errors (Figure 6b, volume > 29). Consequently, the implementation of FFNNs for ANN simulation using samples of a priori knowledge required the development of criteria for assessing their redundancy and limiting the sizes.

### 2.4. Analysis of the Experimental Data

**Figure 7:**
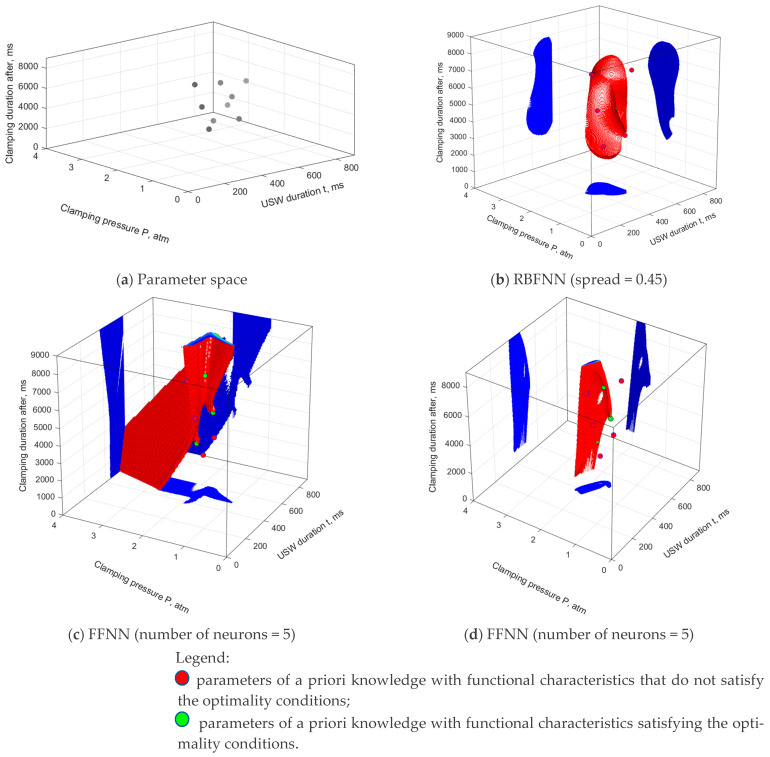
(**a**) The USC parameters of a priori knowledge and the SOP areas, drawn using the (**b**) RBFNN and (**c**,**d**) FFNN models. The learning sample was the experimental data from nine points, according to Table 5.

In the ANN simulation, experimentally measured levels of the functional characteristics of lap joints obtained using the USC parameters presented in Table 4 were applied as a learning sample. The determined values are shown in Table 5, including nine vectors assessed by the Taguchi method. Both RBFNNs (‘goal’ = 0.001) and FFNNs (activation functions—hyperbolic tangent) were implemented. The distribution of the learning sample in the parameter space is shown in Figure 7a, while the SOP areas are presented in Figure 7b–d. As expected, the SOP area, determined using the RBFNN model with an ultra-small sample (Figure 7b), covered the relevant data and tended to the minimum size with lowering of the ‘spread’ parameter. On the one hand, this fact led to more accurate approximation within the range of the experimental USC parameters, but on the other hand it did not allow solving predictive problems. The implementation of the FFNN gave an infinite number of solutions, from completely unrealistic (Figure 7c) to adequate (Figure 7d). Moreover, all these models met the minimum mean square error criterion (the difference between the calculated values and the data of the learning and testing samples). Note that the drawn SOP areas were not limited to the region of the experimental USC parameters and extended into the predicted (extrapolation) area. Nevertheless, general patterns in their behavior were not revealed. This drawback of the ANNs testified to the inefficiency of their use in learning with ultra-small training samples, requiring the use of additional data to limit acceptable solutions in the extrapolation range. The accuracy of the constructed models was estimated according to verification with additional laboratory tests, as described in Section 3.

### 2.5. Analysis of a Posterior Knowledge and Its Implementation

Next, within the framework of the ANN simulation, a posteriori knowledge was added to the a priori knowledge (Table 2). The latter was obtained after or in the process of the experimental investigations and was introduced in sections of the parameter space corresponding to high *t* values but low *τ* levels (Table 6). In this case, the model was limited to knowledge in five sections of the parameter space, which were determined by three values according to Table 4. The learning sample size was 47 vectors of the USC parameters with the corresponding functional characteristics of the lap joints.

**Figure 8:**
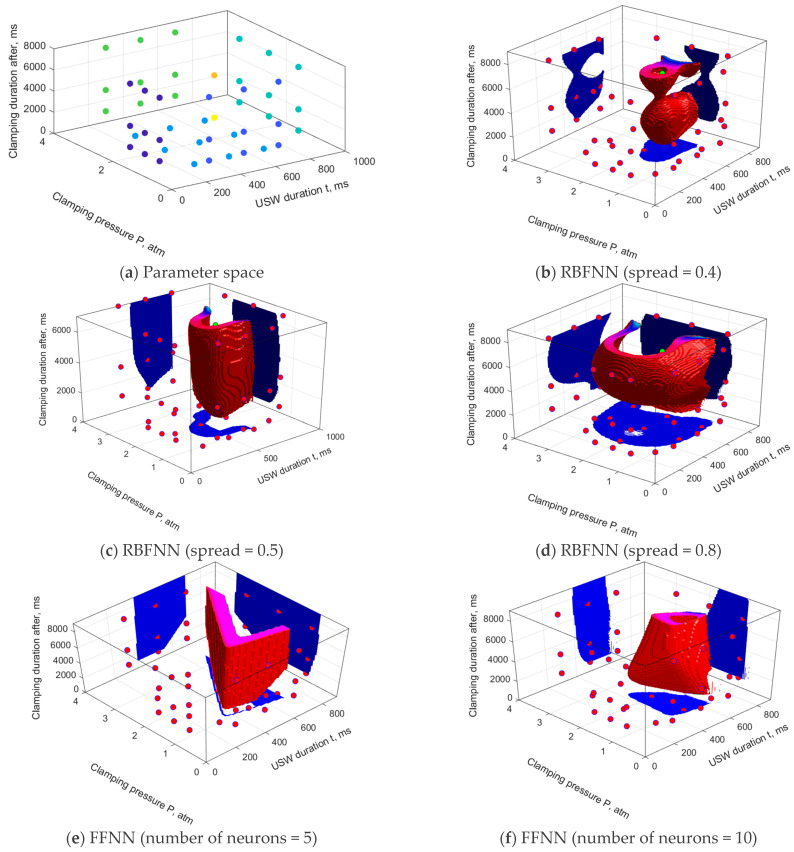

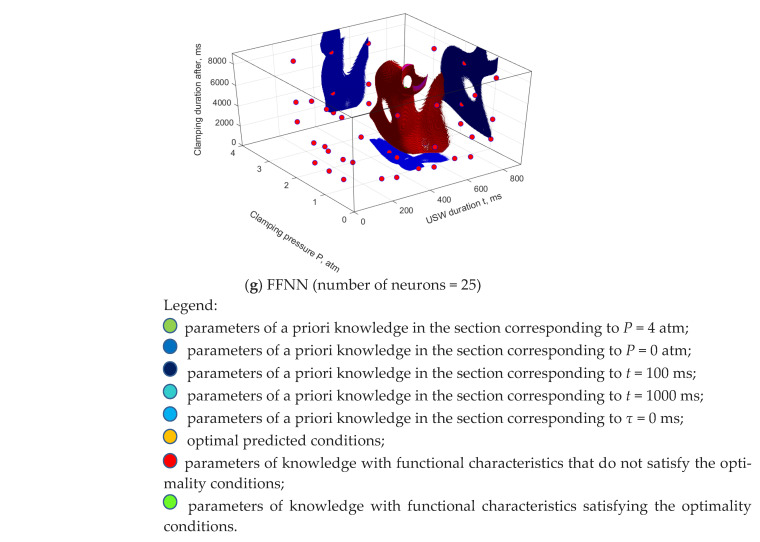
The USC parameters of a priori knowledge (**a**) and the SOP areas, drawn using the RBFNN (**b**–**d**) and FFNN (**e**–**g**) models. The learning sample was both the a priori and a posteriori knowledge.

ANN simulation was carried out implementing both RBFNNs (‘goal’ = 0.001) and FFNNs (activation functions are hyperbolic tangent), as well as using the data from Table 2 and Table 6. A distribution of the learning sample in the parameter space is shown in Figure 8a, while the SOP areas, drawn using various USC parameters, are shown in Figure 8b–g.

Comparing the results obtained using the RBFNNs (Figure 4 and Figure 6), a conclusion was drawn that additional knowledge on the boundary sections could lead both to enhancing the SOP areas (Figure 8b) and their downsizing (Figure 8c,d). Changing the ‘spread’ parameter still affected the sizes of the SOP areas and could be considered as a tool for applying the subjective knowledge of experts.

Enhancing the learning sample size through the use of new knowledge in the FFNN models resulted in the limitation of the SOP areas in the directions of those sections where this new knowledge was determined. In this case, increasing the complexity of the FFNN (through the number of neurons in the hidden layer) played an important role in the non-linearity of the model: from a simple quasi-linear law (Figure 8e) to a significantly non-linear one (Figure 8g). Unlike the RBFNN models, the way to express non-formalized knowledge in the FFNN ones was to justify the number of neurons (*N*) in the hidden layer. In this example, the model had to be considered as coarse (undertrained) at *N* = 5, displaying an unreasonably complex law (overtrained) at *N* = 25, but adequate at *N* = 10.

**Figure 9:**
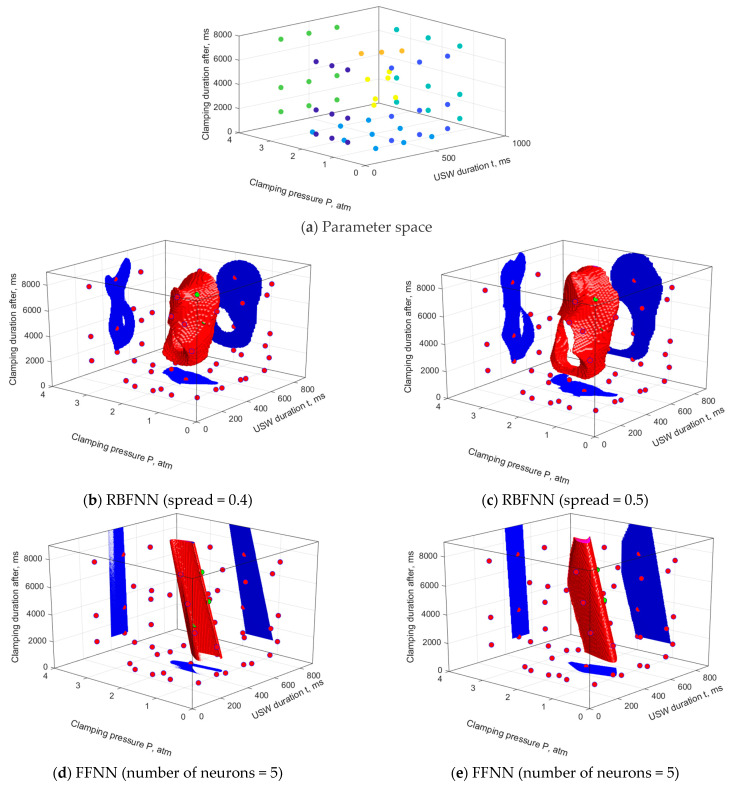

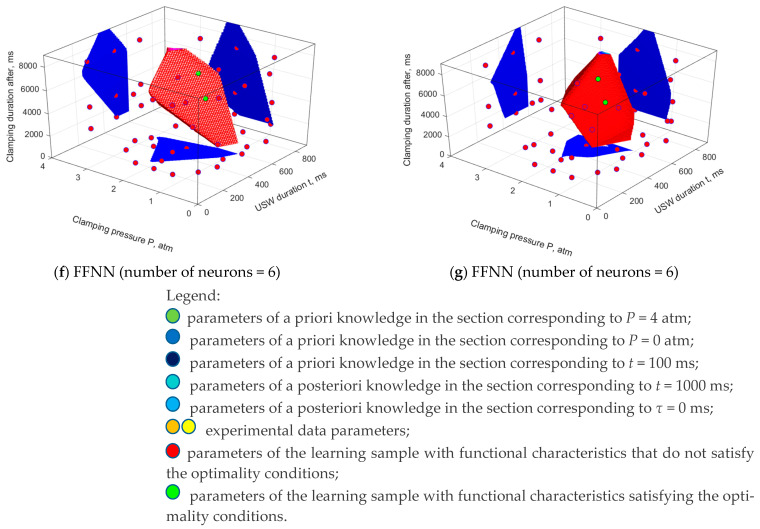
The USC parameters of both knowledge and experimental data (**a**), the SOP areas drawn using the RBFNN (**b**,**c**) and FFNN (**d**–**g**) models. The learning sample was the experimental data, as well as both a priori and a posteriori knowledge.

Subsequent ANN simulation was performed using a sample that included nine experimentally measured values as well as both a priori and a posteriori knowledge about the USC parameters. The total sample size was 54 vectors. A distribution of the experimental parameters in the search area is presented in Figure 9a. The SOP areas drawn using the RBFNN models with different values of the ‘spread’ parameter are presented in Figure 9b,c, while Figure 9d reflects the case for the FFNN.

As a result of comparing the data presented in Figure 8 and Figure 9, the following conclusions were drawn about the impact of the addition of both a priori and a posteriori knowledge on the assessment of the SOP areas:

1. The RBFNN models became more complex. The sizes of the SOP areas increased, including in the extrapolation region.
2. The SOP areas determined using the FFNN models were limited to the extrapolation regions and acquired a general pattern (the uncertainty of the behavior decreased). However, this phenomenon did not solve the problem of multiple solutions.
3. The models drawn with the addition of both a priori and a posteriori knowledge should not be considered as competing, due to their imprecise expression. SOP areas drawn both with and without them had to be jointly analyzed.
4. The problem of choosing the learning sample size of both a priori and a posteriori knowledge had to be justified as a problem of finding the optimal size according to the minimum extrapolation error criterion.

## 3. Verification of the Results of ANN Simulation

Verification of the results of the ANN simulation was carried out using USC parameters (*t* = 510 ms, *τ* = 9000 ms, *P* = 1.85 atm) from the extrapolation region, for which the predicted values (i) varied most significantly, (ii) could be implemented using the available USW facility, and (iii) were located in the direction of the greatest expected errors of the ANN simulation. Table 7 presents the predicted values of the functional characteristics of the USC lap joints, obtained using the predicted USC parameters:

- the RBFNNs were trained with only the experimental data (the ‘RBFNN’ row in Figure 8b) and with both a priori and a posteriori knowledge in addition to the experimental data (the ‘RBFNN (+ knowledge)’ row in Figure 9c);
- for the FFNNs, due to the ambiguity of their training, the predicted values were calculated from a variety of the results (examples shown in Figure 8c,d and Figure 9d,e) after training with only the experimental data (the ‘FFNN’ row) and with both a priori and a posteriori knowledge in addition to the experimental data (the ‘FFNN (+ knowledge)’ row).

**Figure 10:**
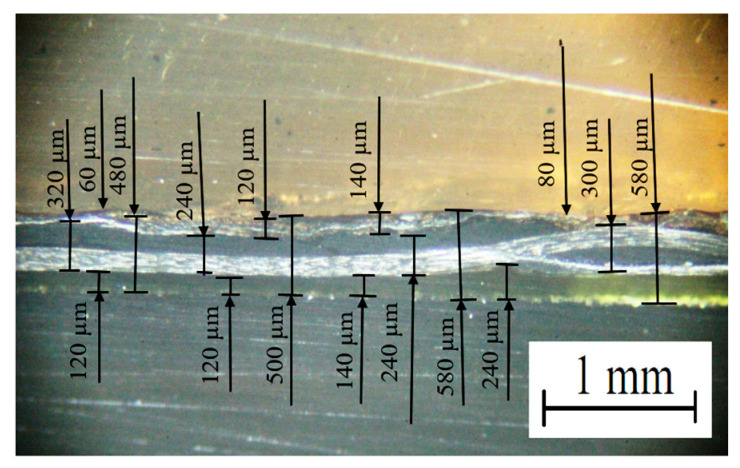
The optical image of the cross section of the lap joint obtained using the optimal USC parameters.

Table 7 also includes the experimentally measured values of the functional characteristics of the lap joint, obtained using the optimal USC parameters. Figure 10 shows an optical image of its cross-section. The USC lap joint thinning was 330 ± 10 µm, while the top ED was noticeably (but not uniformly) thinned (*δ*_ED top_ = 80 ± 60 µm). At the same time, the bottom ED thickness was changed to a much lesser extent (*δ*_ED bottom_ = 170 ± 70 µm). The “CF fabric layer” thickness was 280 ± 40 µm, which was slightly greater than the initial prepreg value (considering possible scattering). Samples of such USC lap joints fractured at rather high stress levels of 62.60 ± 3.13 MPa and values of elongation at the break of 4.22 ± 0.21%.

An analysis of the results presented in Table 7 showed that the RBFNN model trained with both additional a priori and a posteriori knowledge significantly increased its accuracy, so the predicted and experimentally measured values were closer to each other. This fact confirmed the possibility of using the RBFNN models for prediction by extrapolation using ultra-small samples after artificial expansion of their sizes. Respectively, such models should be considered as the most promising ones.

On the other hand, the FFNN models, both with additional a priori and a posterior knowledge and without them, were characterized by average predicted values close to the real ones. However, the scatter of the predicted values with additional knowledge in the learning samples increased significantly (Table 7), bringing into question the applicability of this approach. Additional research is required to determine the reasons for these variations.

A comparison of the predicted and real values (Table 6) showed that mechanical properties were achieved that exceeded the experimental data obtained above (mode 6). In this case, the *δ*_ED top_ and *δ*_ED+CF_ parameters were outside the acceptable ranges according to Table 1. Nevertheless, these values reflected the optimal USC parameters, enabling improvement of the mechanical properties (falling within the range specified in Table 1), a situation which was not obtained using modes 1–9. On this basis, it was decided to change the SOP areas according to Table 8.

**Figure 11:**
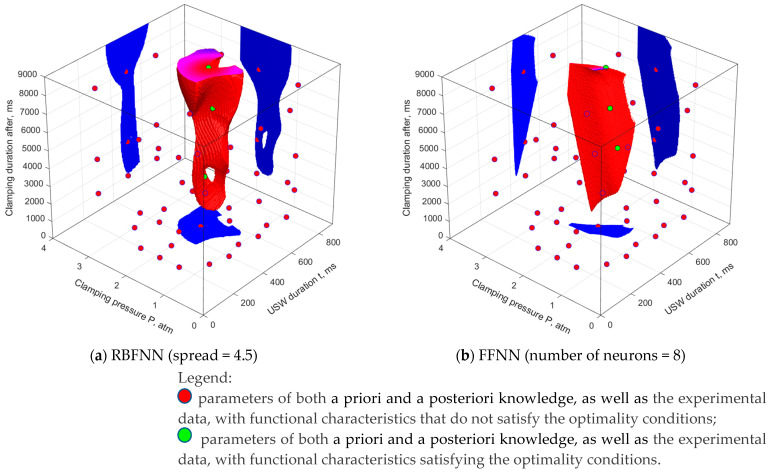
The USC parameter space and the SOP areas, drawn using the RBFNN (**a**) and FFNN (**b**) models. The learning sample was the (1–9, ‘optimal’) experimental data as well as both a priori and a posteriori knowledge.

The results of ANN simulation using the updated models trained with both a priori and a posteriori knowledge in addition to the experimental data (Table 5, modes 1–9; Table 7, the optimal USC parameters) are summarized in Figure 11.

## 4. Discussion

As noted above, most researchers of USW/USC procedures implemented for joining composites based on thermoplastic binders have focused on laminates. The reason for this includes both their high strength properties and the practical relevance of the obtained results. Similar, ANNs have been applied for solving such problems [12]. However, the key advantage of USC procedures is their short duration, expanding the application areas. For example, data on USW patterns concerning particulate composites based on thermoplastic matrices have been reported [66,67,68], in addition to which the results presented above could be adapted to develop such procedures for composites fabricated from polymer blends or hybrid polymer mixtures [69,70,71]. In this way, ANN simulation performed to optimize the USW parameters for obtaining USC ‘PEI adherend/Prepreg (CF-PEI fabric)/PEI adherend’ lap joints enabled an understanding of their complex mutual influence on the functional characteristics.

By analogy with the approach implemented for manufacturing laminates from sequential layers of both thermoplastic and CF fabric [72], a similar material was fabricated from PEI/CF prepregs and EDs in this study. To achieve this goal, the optimal USC parameters were applied, which were determined through ANN simulation. The method for manufacturing the PEI-impregnated PEEK-based prepreg based on the CF fabric (Toray Cetex TC1200, Toray Industries, Japan) was described in a previous paper by the authors [38].

**Figure 12:**
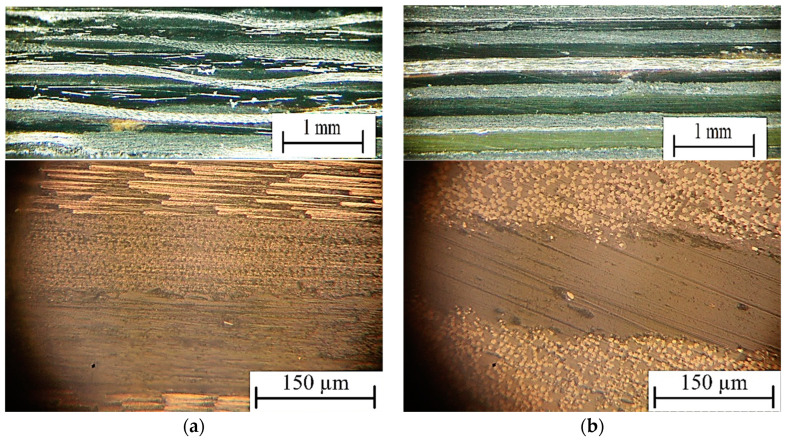
Optical images of the cross sections of the five-layer USC laminates of (**a**) PEI-impregnated and (**b**) PEEK-based CF fabric-based prepregs.

Figure 12 shows cross-sections of the laminates made from (a) PEI-impregnated and (b) commercially available PEEK-based prepregs. The USC parameters justified above were used (clamping pressure of 1.85 atm, USW duration of 510 ms, clamping duration after USW of 9000 ms). The thickness of the PEI prepreg was 250 ± 20 µm, while that of the PEEK-based one was 170 ± 20 µm (PEEK prepregs were US-consolidated without EDs). The number of prepreg layers was five, while it was four for the EDs (for the PEI prepregs only). The USC procedures were carried out using the layer-by-layer method. The total number of passes of the USC instrument (sonotrode) was four. Under such conditions, it was possible to form non-porous USC laminates (joints) with satisfactory quality (minimal damage to the components). Thereby, the correctness of the optimal USW parameters predicted by ANN simulation was verified experimentally. This approach can also be implemented to repair damaged regions of other composites based on thermoplastic binders.

The research areas in which USC procedures have been implemented to form fiber-reinforced composites based on thermoplastic binders were already mentioned above:

- continuous ultrasonic impregnation and consolidation of thermoplastic matrix composites;
- ultrasonic-assisted consolidation of commingled thermoplastic/glass fiber rovings;
- consolidation of composite pipes by in situ ultrasonic welding (thermoplastic matrix composite tape);
- ultrasonic vibration-assisted automated fiber placement;
- automated fiber placement and tape laying (thermoplastic composite prepreg);
- filament winding and automated fiber placement with in situ consolidation.

All of these were characterized by the use of different USC parameters. Moreover, the sizes of the experimental data samples were very limited. Respectively, the authors believe that the approach developed in this study, based on ANN simulation using ultra-small samples, is of undoubted practical interest and can be applied to solve related problems, including the automated tape placement [73,74].

## 5. Conclusions

1. By ANN simulation, an approach was developed to establish the relationship between the USC parameters and the functional characteristics of ‘PEI adherend/Prepreg (CF-PEI fabric)/PEI adherend’ lap joints. For this purpose, the RBFNN and FFNN models were tested and the most reliable results were selected.
2. The real values of the functional characteristics of the USC joints were measured experimentally and used to train the ANNs. For improving the effectiveness, the experimental sample was significantly expanded by adding both a priori and a posteriori knowledge.
3. The conducted studies into the influence of the ANN simulation parameters and the addition of both a priori and a posteriori knowledge to the learning sample on the accuracy of the drawn SOP areas showed the following outcomes. The RBFNN model trained using the sample with the additional a priori and a posteriori knowledge significantly increased its accuracy, and the predicted data came closer to the experimental results. This fact confirmed the possibility of using RBFNN models for prediction through extrapolation of ultra-small samples after artificial expansion of their sizes. On the other hand, the FFNN models, even without adding both a priori and a posteriori knowledge, gave average values close to the real ones. However, the scatter of the predicted values increased significantly after the addition of both a priori and a posteriori knowledge, so the obtained results sow doubt on the applicability of this approach.
4. The results of ANN simulation were verified, since the implementation of the predicted USC parameters made it possible to maintain the structural integrity of the reinforcing CF fabric and ensure the maximum strength properties.
5. The results of ANN simulation for optimizing the USC parameters were suitable for manufacturing the ‘PEI adherend/Prepreg (CF-PEI fabric)/PEI adherend’ laminates (and those from the commercial PEEK/CF prepregs). Such procedures enable the formation of an even (regular) structure characterized by a minimum number of discontinuities and minimal damage to the joined components.

## Figures and Tables

**Table 1:** The acceptable ranges of the functional characteristics of the USW joints.

Property	Range
Tensile strength, MPa	30 ≤ *σ* ≤ 65
Elongation at break, %	1.75 ≤ *ε* ≤ 4.50
USC joint thinning, μm	100 ≤ Δ*d* ≤ 400
Top ED thickness, μm	120 ≤ *δ*_ED top_ ≤ 180
Distance between PEI adherends, μm	450 ≤ *δ*_ED+CF_ ≤ 550
Top PEI adherend integrity, +/–	0 ≤ *C*

**Table 2:** A priori knowledge, as well as predicted USC parameters and functional characteristics of the lap joints.

Conditions	Parameters			Characteristics					
	USW Duration, ms	Clamping Pressure, atm	Duration of Clamping after USW, ms	σ, MPa	*ε*, %	Δ*d,* μm	*δ*_ED top_, μm	*δ*_ED + CF_, μm	*C*
1. Optimal predicted properties	500	1.5	3000	65	4.5	400	120	450	1
	500	1.5	7000						
2. Constancy of dimensions	100	1÷2	1000 ÷ 7000	0	0	0	250	750	1
	300 ÷ 700	4	1000 ÷ 7000						
	300 ÷ 700	0.5	1000 ÷ 7000						

**Table 3:** The errors of ANN simulation using a priori knowledge learning samples.

Sample Size	MSE for Learning Sample	Number of Neurons (*N*)	Standard Deviation		
			Analysis Region	Approximation Region	Extrapolation Region
	RBFNN (goal = 1 × 10^–3^)				
20	9.72 × 10^−4^	10	0.0636	0.0336	0.047
29	2.3691 × 10^−4^	20	0.0318	0.0194	0.0189
38	4.1871 × 10^−5^	20	0.0191	0.0069	0.0139
56	2.6309 × 10^−4^	18	0.0236	0.0224	0.0173
	RBFNN (goal = 1 × 10^–6^)				
20	7.22 × 10^−7^	16	0.0345	0.0399	0.0242
29	2.852 × 10^−31^	27	0.0074	6.0390 × 10^−4^	0.0025
38	2.6257 × 10^−10^	34	0.0074	6.0496 × 10^−4^	0.0025
56	2.6669 × 10^−7^	37	0.0077	5.6014 × 10^−4^	0.0033
	FFNN (average of seven models)				
20	7.71 × 10^−6^	6	0.1395	0.0470	0.0905
29	9.45 × 10^−7^	6	0.0325	0.0001	0.0134
38	6.79 × 10^−6^	6	0.0210	0.0060	0.0979
56	6.68 × 10^−6^	6	0.3247	0.0002	0.2165

**Table 4:** The UCC parameters obtained by combining the factors and their levels in the L9 format, according to the Taguchi method.

Experiment No.	Parameters		
	USW Duration, ms	Clamping Pressure, atm	Duration of Clamping after USC, ms
(1)	400	1.5	3000
(2)	400	1.7	5000
(3)	400	1.9	7000
(4)	500	1.5	5000
(5)	500	1.7	7000
(6)	500	1.9	3000
(7)	600	1.5	7000
(8)	600	1.7	3000
(9)	600	1.9	5000

**Table 5:** The functional characteristics of the joints obtained using the USC parameters presented in Table 4.

No.	Tensile Strength (*σ*), MPa	Elongation at Break (*ε*), %	USC Joint Thinning (Δ*d*), μm	Top ED Thickness (*δ*_ED top_), μm	Bottom ED Thickness (*δ*_ED bottom_), μm	Mean ED Thickness (*δ*_ED mean_), μm	Distance between PEI Adherends (*δ*_ED+CF_), μm	“CF layer” Thickness (*δ*_CF_), μm	Top PEI Adherend Integrity, +/–
1	26.0 ± 1.6	1.61 ± 0.05	110 ± 60	140 ± 40	220 ± 40	180 ± 80	620 ± 20	230 ± 30	+
2	24.7 ± 1.7	1.41 ± 0.06	160 ± 30	160 ± 60	200 ± 40	170 ± 70	620 ± 40	230 ± 70	+
3	25.0 ± 1.5	1.70 ± 0.07	230 ± 20	170 ± 30	180 ± 40	180 ± 40	540 ± 40	210 ± 50	+
4	35.2 ± 1.4	2.07 ± 0.07	160 ± 20	150 ± 70	160 ± 40	150 ± 70	540 ± 40	190 ± 70	+
5	46.2 ± 3.2	2.85 ± 0.10	120 ± 20	150 ± 150	115 ± 65	150 ± 150	550 ± 50	250 ± 110	+
6	61.3 ± 3.1	3.81 ± 0.11	400 ± 40	160 ± 40	175 ± 75	175 ± 75	500 ± 40	190 ± 70	+
7	34.3 ± 2.1	2.00 ± 0.07	360 ± 20	90 ± 70	130 ± 110	130 ± 110	420 ± 80	200 ± 60	–
8	38.2 ± 2.3	2.12 ± 0.07	290 ± 20	110 ± 50	170 ± 50	140 ± 80	490 ± 70	210 ± 30	–
9	41.6 ± 1.7	2.37 ± 0.08	420 ± 20	100 ± 60	170 ± 70	140 ± 100	420 ± 60	180 ± 40	–

**Table 6:** A posteriori knowledge about the functional characteristics of the USC lap joints.

Conditions	Parameters			Properties					
	USW Duration, ms	Clamping Pressure, atm	Duration of Clamping after USC, ms	σ, MPa	*ε*, %	Δ*d,* μm	*δ*_ED top_, μm	*δ*_ED + CF_, μm	*C*
Failure of lap joints during USC (before testing)	900	1 ÷ 3	1000÷7000	40	1.5	400	50	350	−1
Negligible strength due to minimum clamping duration after USC	300	1 ÷ 3	0	10	0.75	50	250	650	1
	500	1 ÷ 3				200	160	550	1
	700	1 ÷ 3				400	50	350	−1

**Table 7:** The functional characteristics of the USC lap joints.

No.	*σ*, MPa	*ε*, %	Δ*d,* μm	*δ*_ED top_, μm	*δ*_ED + CF_, μm	*C*
RBFNN	−3.52	0.12	661.24	89.86	316.53	−1.04
RBFNN (+knowledge)	41.17	2.29	98.08	191.29	551.46	0.91
FFNN	31.70 ± 6.93	2.06 ± 0.58	268.3 ± 146.0	164.6 ± 5.0	533.9 ± 53.4	1.0 ± 0.0
FFNN (+knowledge)	45.50 ± 15.78	2.9 ± 0.9	208.3 ± 208.3	148.5 ± 94.9	567.04 ± 160.50	0.88 ± 0.10
Optimal	62.60 ± 3.13	4.22 ± 0.21	330 ± 10	80 ± 60	280 ± 40	1

**Table 8:** The acceptable ranges of the functional characteristics of the USC joints, justified by ANN simulation and verification of its results.

Property	Range
Tensile strength, MPa	30 ≤ *σ* ≤ 65
Elongation at break, %	1.75 ≤ *ε* ≤ 4.50
USC lap joint thinning, μ m	100 ≤ ∆*d* ≤ 400
Top ED thickness, μ m	65 ≤ *δ*_ED top_ ≤ 180
Distance between PEI adherends, μ m	230 ≤ *δ*_ED+CF_ ≤ 550
Top PEI adherend integrity, +/–	0.0 ≤ *C*

## Data Availability

Data are contained within the article.
